# Vaccination against type 1 angiotensin receptor prevents streptozotocin-induced diabetic nephropathy

**DOI:** 10.1007/s00109-015-1343-6

**Published:** 2015-09-26

**Authors:** Dan Ding, Yimei Du, Zhihua Qiu, Sen Yan, Fen Chen, Min Wang, Shijun Yang, Yanzhao Zhou, Xiajun Hu, Yihuan Deng, Shijia Wang, Liangping Wang, Hongrong Zhang, Hailang Wu, Xian Yu, Zihua Zhou, Yuhua Liao, Xiao Chen

**Affiliations:** 1 Laboratory of Cardiovascular Immunology, Key Laboratory of Molecular Targeted Therapies of the Ministry of Education, Institute of Cardiology, Union Hospital, Tongji Medical College of Huazhong University of Science and Technology, Wuhan, 430022 China; 2 Fuwai Hospital, Peking Union Medical College and Chinese Academy of Medical Sciences, Beijing, China

**Keywords:** Angiotensin receptor, Streptozotocin, Renin-angiotensin system, Diabetic nephropathy, Vaccine

## Abstract

**Abstract:**

Recently, our group has developed a therapeutic hypertensive vaccine against angiotensin (Ang) II type 1 receptor (AT1R) named ATRQβ-001. To explore its potential effectiveness on streptozotocin-induced diabetic nephropathy, male Sprague Dawley rats were randomly divided into two groups: a control and a diabetic model. After 1 week, the diabetic rats were divided into four subgroups (each with 15 rats) for 14-week treatments with saline, olmesartan, ATRQβ-001, and Qβ virus-like particle (VLP), respectively. In addition to lower blood pressure, ATRQβ-001 vaccination ameliorated biochemical parameter changes of renal dysfunction, mesangial expansion, and fibrosis through inhibiting oxidative stress, macrophage infiltration, and proinflammatory factor expression. Furthermore, ATRQβ-001 vaccination suppressed renal Ang II-AT1R activation and abrogated the downregulation of angiotensin-converting enzyme 2-Ang (1–7), similar to olmesartan treatment, while no obvious feedback activation of circulating or local renin-angiotensin system (RAS) was only observed in vaccine group. In rat mesangial cells, the anti-ATR-001 antibody inhibited high glucose-induced transforming growth factor-β1 (TGF)-β1/Smad3 signal pathway. Additionally, no significant immune-mediated damage was detected in vaccinated animals. In conclusion, the ATRQβ-001 vaccine ameliorated streptozotocin-induced diabetic renal injury via modulating two RAS axes and inhibiting TGF-β1/Smad3 signal pathway, providing a novel, safe, and promising method to treat diabetic nephropathy.

**Key messages:**

- Overactivation of RAS plays a crucial role in the development of the DN.
- Our aim was to verify the effectiveness of ATRQβ-001 vaccine in STZ-induced DN.
- The ATRQβ-001 modulated two RAS axes and inhibited TGF-β1/Smad3 signal pathway.
- The vaccine therapy may provide a novel, safe, and promising method to treat DN.

**Electronic supplementary material:**

The online version of this article (doi:10.1007/s00109-015-1343-6) contains supplementary material, which is available to authorized users.

## Introduction

Diabetic nephropathy (DN) is the most common cause of end-stage renal diseases, responsible for over 40 % of all cases in the USA, and this number is likely to increase unabated [1]. Overactivation of the renin-angiotensin system (RAS) plays a crucial role in the development of the disease [2]. Current treatment, aimed at slowing progression, concentrates on two inter-related therapeutic strategies: blood pressure reduction and blockade of the RAS [3–5]. Such treatments have been shown to reduce the functional changes seen in DN and also to attenuate the structural abnormalities that characterize this disease [6–8]. At present, blockade of the RAS is achieved by two major drug classes: angiotensin-converting enzyme (ACE) inhibitors and angiotensin II (Ang II) receptor blockers (ARBs), agents that also lower systemic blood pressure [9]. However, they are not completely effective in preventing or reversing the progress of DN, in part due to a compensatory increase in plasma renin activity (PRA) or Ang II [10]. Current therapies are insufficient, necessitating the search for new therapeutic strategies in DN.

Recently, we developed a therapeutic hypertensive vaccine ATRQβ-001, a peptide (ATR-001) derived from human Ang II receptor type 1 (AT1R) conjugated with Qβ bacteriophage virus-like particles, which decreased the blood pressure of hypertensive animals effectively through diminishing the pressure response and inhibiting signal transduction initiated by Ang II with no obvious feedback activation of circulating or local RAS [11]. In our previous study, we demonstrated that an epitope from the rat AT1R, designated as ATR12181, could decrease SBP of spontaneously hypertensive rats (SHRs) and provide excellent protections in target organs [12]. Following our work, Hiroshi Itoh and his colleagues emphasized vaccination against AT1R for the prevention of L-NAME-induced nephropathy in SHRs, not only for the attenuation of hypertension [13]. Ang II and other components of RAS also have a central role in the pathogenesis and progression of diabetic renal injuries. Accordingly, this study was designed to explore the possibility of the ATRQβ-001 vaccine in ameliorating of experimental DN and the potential mechanisms. The study was undertaken in Sprague Dawley rats treated with streptozotocin that developed renal injury similarities to human DN [14].

## Methods

Detailed methods are available in the Online Supplement.

### Animals

Male Sprague Dawley rats weighing 200–250 g were purchased from the experimental animal research center (Hubei Province, China). All animals were kept in the pathogen-free room in the experimental animal center (Tongji Medical College of Huazhong University of Science and Technology, Wuhan, China), and all experiments were carried out in accordance with guidelines for the Care and Use of Laboratory Animals (Science and Technology Department of Hubei Province, China, 2005). Experimental diabetes was induced by intraperitoneal injection of the β cell toxin streptozocin (60 mg/kg) dissolved in fresh sodium citrate buffer (pH 4.5) following an overnight fast. Animals with plasma glucose concentrations in excess of 16.7 mmol/l, 1 week postinduction of diabetes, were included in the study. Sham-injected control animals (sodium citrate buffer, pH 4.5) were followed concurrently. Diabetes rats were then randomized into four groups (*n* = 15), receiving one of the following treatments: (1) DN group: equal volume saline injection subcutaneously (s.c); (2) OM group: olmesartan, 5 mg/kg/day via oral gavage; (3) ATRQβ-001group: the ATRQβ-001 vaccine, which was immunized s.c 400 μg on days 0, 14, and 21, formulated in aluminum hydroxide gel; (4) virus-like particle (VLP) group: 400μg Qβ VLP injected as group 3. Each week, rats were weighed and their blood glucose levels were measured. ATR-001-specific antibody titers were detected in every 2 weeks. Every 4 weeks, systolic blood pressure (SBP) was determined in preheated conscious rats via tail-cuff plethysmography using a non-invasive blood pressure controller and PowerLab system.

### Plasma renin activity, Ang II, and Ang (1–7) concentration measurement

The rats were decapitated between 9 am and 12 am. Blood samples were collected and divided into two parts: one was mixed with the enzyme inhibitor mixture (1 ml blood in 50 μl inhibitor mixture including 20 μl 0.3 mol/l EDTA, 10 μl 0.32 mol/l dimercaprol, and 20 μl 0.34 mol/l 8-OH-quinoline sulfate) for PRA and Ang II concentration measurement by radioimmunoassay (RIA) according to the assay kit instruction (NIBT, Beijing); the other was used for biochemical experiments. For tissue Ang II and Ang (1–7) measurement, immediately after harvesting and weighing, the kidney cortex were immersed in ice-cold methanol, minced, and homogenized with tissue homogenizers. The homogenates were centrifuged (4000 rpm, 4 °C, 15 min), and the supernatants were dried overnight in a vacuum centrifuge. The dried residue was reconstituted in 1 ml RIA buffer and then was subjected to HPLC to separate Ang II from other substances. The tissue Ang II concentration was detected according to the assay kit instruction (NIBT, Beijing), and Ang (1–7) was measured by HPLC.

### Biochemical measurement

The plasma samples were used for the measurement of glucose, lipid level, creatinine (Cr), and blood urea nitrogen (BUN). Urine samples were collected using metabolic cages, and the supernatant was used for examination of the 24-h urinary protein.

### Histopathology

Parts of fresh left renal cortex were immediately fixed in 0.25 % glutaraldehyde for transmission electron microscopy (TEM). The other parts were fixed with 4 % paraformaldehyde overnight, embedded in paraffin for histopathology. Sections were stained with hematoxylin-eosin (H&E), Masson’s trichrome, and periodic acid-Schiff (PAS). Frozen sections were stained with dihydroethidium (DHE).

### Immunohistochemistry

Immumohistochemical staining was performed to detect the expression of TGF-β1 (1:100, Everest Biotech) and macrophages (CD68, 1:100, AbD Serotec) in glomeruli.

### Immunofluorescence

Immunofluorescence staining of nephrin and podocin expression was performed on the paraffin sections of kidneys using monoclonal anti-nephrin (1:200, Abcam) and anti-podocin antibodies (1:200, Abcam).

### Cell culture and treatment

Rat mesangial cells (RMCs) were cultured in Dulbecco’s modified Eagle’s medium supplemented with 10 % fetal bovine serum, 100 U/ml penicillin, and 100 mg/ml streptomycin at 37 °C in 95 % air and 5 % CO_2_. Cells plated on 60-mm dishes were cultured to 80 % confluence and divided into five groups: control group, in which cells were incubated in 5 mmol/l D-glucose DMEM, and 20 mmol/l D-mannitol was added to the medium in order to take into the account of the effect of high osmolarity in other cell groups; HG group, in which cells were stimulated with a high concentration of glucose (25 mmol/l) only for 24 h; Los group, in which cells were pretreated with 10^−6^ mol/l losartan for 1 h; anti-ATR-001 group, in which cells were pretreated with anti-ATR-001 for 1 h; anti-NATR-001 group, in which cells were pretreated with anti-NATR-001 for 1 h. All cells except control group received stimulation with a high concentration of glucose (25 mmol/l) for 24 h after treatment. Cell protein and messenger RNA (mRNA) were extracted for Western blot and qRT-PCR analysis.

### Statistical analysis

Data were shown as the mean ± SEM. Statistical analyses of the data were performed with one-way ANOVA using SPSS18.0. *P* < 0.05 was considered statistically significant.

## Results

### Animal characteristics

In comparison with control animals, diabetic rats had significantly reduced body weight, which was unaffected by treatment. The ratio of kidney weight/body weight was increased, while ATRQβ-001 vaccine and olmesartan treatments decreased the level. Blood glucose and lipid levels were elevated to a similar extent in all diabetic rat groups, irrespective of treatment (Table 1). No evidence of skin damages at the site of subcutaneous injection was noted in vaccine-treated animals. Less activities and poorer hair were shown in diabetic rats, but the reactivity was the same as normal rats.

**Table 1:** Animal characteristics

	Con	DN	VLP	OM	ATRQβ-001
Body weight (g)	359 ± 12.69	173.3 ± 9.78*	169.2 ± 8.47*	216 ± 18.68*	184.7 ± 9.7*
Kidney weight (g)	1.19 ± 0.03	1.15 ± 0.03	1.14 ± 0.02	1.36 ± 0.08	1.11 ± 0.04
KW/BW (%)	0.33 ± 0.01	0.67 ± 0.02*	0.69 ± 0.02*	0.58 ± 0.02*^#^&	0.63 ± 0.03*^#^
Blood glucose (mmol/l)	7.8 ± 1.84	31.6 ± 3.11*	34.0 ± 1.84*	30.4 ± 1.30*	30.2 ± 2.55*
TC (mmol/l)	1.49 ± 0.1	2.48 ± 0.56*	2.6 ± 0.63*	2.33 ± 0.16*	2.58 ± 0.34*
TG (mmol/l)	0.85 ± 0.19	2.53 ± 0.28*	2.32 ± 0.17*	2.19 ± 0.37*	2.31 ± 0.28*
LDL-C (mmol/l)	2.87 ± 0.44	4.48 ± 0.65*	4.21 ± 0.71*	4.09 ± 0.75*	4.01 ± 0.91*
HDL-C (mmol/l)	0.77 ± 0.04	0.81 ± 0.13	0.79 ± 0.19	0.96 ± 0.11	0.98 ± 0.05

Values are mean ± SEM

*KW/BW* kidney weight/body weight, *TC* total cholesterol, *TG* total triglyceride, *LDL-C* low-density lipoprotein cholesterol, *HDL-C* high-density lipoprotein cholesterol, *Con* control group, *DN* diabetes with saline treatment, *VLP* diabetes with Qβ VLP treatment, *OM* diabetes with olmsartan treatment, *ATRQβ-001* diabetes with the ATRQβ-001 vaccine treatment

*P < 0.05 vs. Con; #*P* < 0.05 vs. DN; &*P* < 0.05 vs. VLP

### ATRQβ-001 vaccination effectively reduced blood pressure and reversed biochemical parameters of renal dysfunction in STZ-induced diabetic rats

**Fig. 1:**
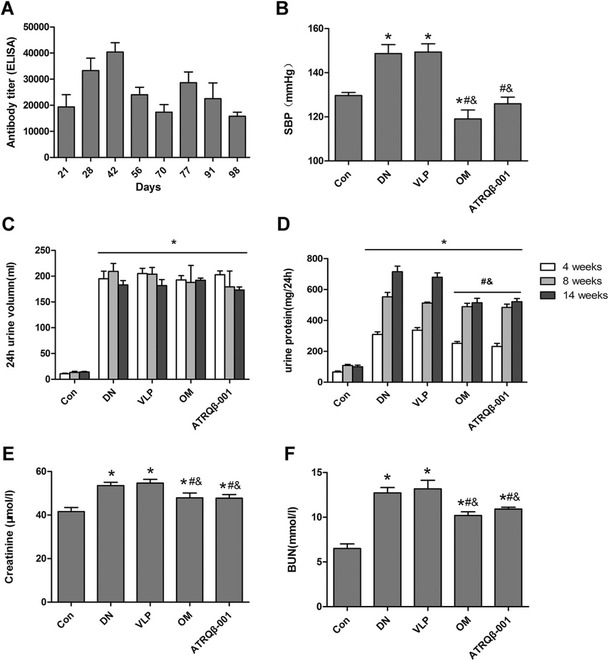
Antibody titers, systolic blood pressure, and biochemical parameters. **a** Diabetic rats were immunized on days 0, 14, 21, and 70, and the ATR-001-specific antibody titers were screened on days 21, 28, 42, 56, 70, 77, 91, and 98. **b** Systolic blood pressure on weeks 14; *P < 0.05 vs. the control group; #*P* < 0.05 vs. DN group; &*P* < 0.05 vs. VLP group. **c** 24 h urine volume; *P < 0.05 vs. the control group at the same time point. **d** 24 h urine protein; *P < 0.05 vs. the control group at the same time point; #*P* < 0.05 vs. DN group at the same time point; &*P* < 0.05 vs. VLP group at the same time point. **e** Plasma creatinine level (*n* = 10). **f** Blood urea nitrogen level (*n* = 10). *P < 0.05 vs. control group; #*P* < 0.05 vs. DN group; &*P* < 0.05 vs. VLP group

To examine antibody production, we measured the amounts of anti-ATR-001 antibodies produced in response to vaccination with ATRQβ-001. After the second injection of the vaccine, the ATR-001-specific antibody titer was 1:10,000 to 1:30,000, and it rose after the third injection, then peaked on day 42 (1:40,000) and gradually decreased thereafter (Fig. 1a). To investigate the efficacy in blood pressure, systolic blood pressure (SBP) levels were measured by the tail-cuff method. Over the course of the study, SBP was elevated in DN compared to normal rats, while rats immunized with the ATRQβ-001 vaccine were decreased compared to the VLP group, with a maximum decrease of 19.4 mmHg (129.9 + 2.9 vs. 149.3 + 3.6 mmHg, *P* < 0.01, Fig. 1b).

To evaluate the biochemical parameters of renal function, 24 h urine volume, and total protein excretion, blood urea nitrogen and creatinine were measured. Diabetic rats exhibited higher levels in all these parameters. ATRQβ-001 vaccine and olmesartan-treated rats significantly reduced these parameters compared to DN and VLP groups, and no significant differences were between these two groups (Fig. 1c–f).

### ATRQβ-001 vaccination prevented podocyte injury and loss as well as mesangial expansion

**Fig. 2:**
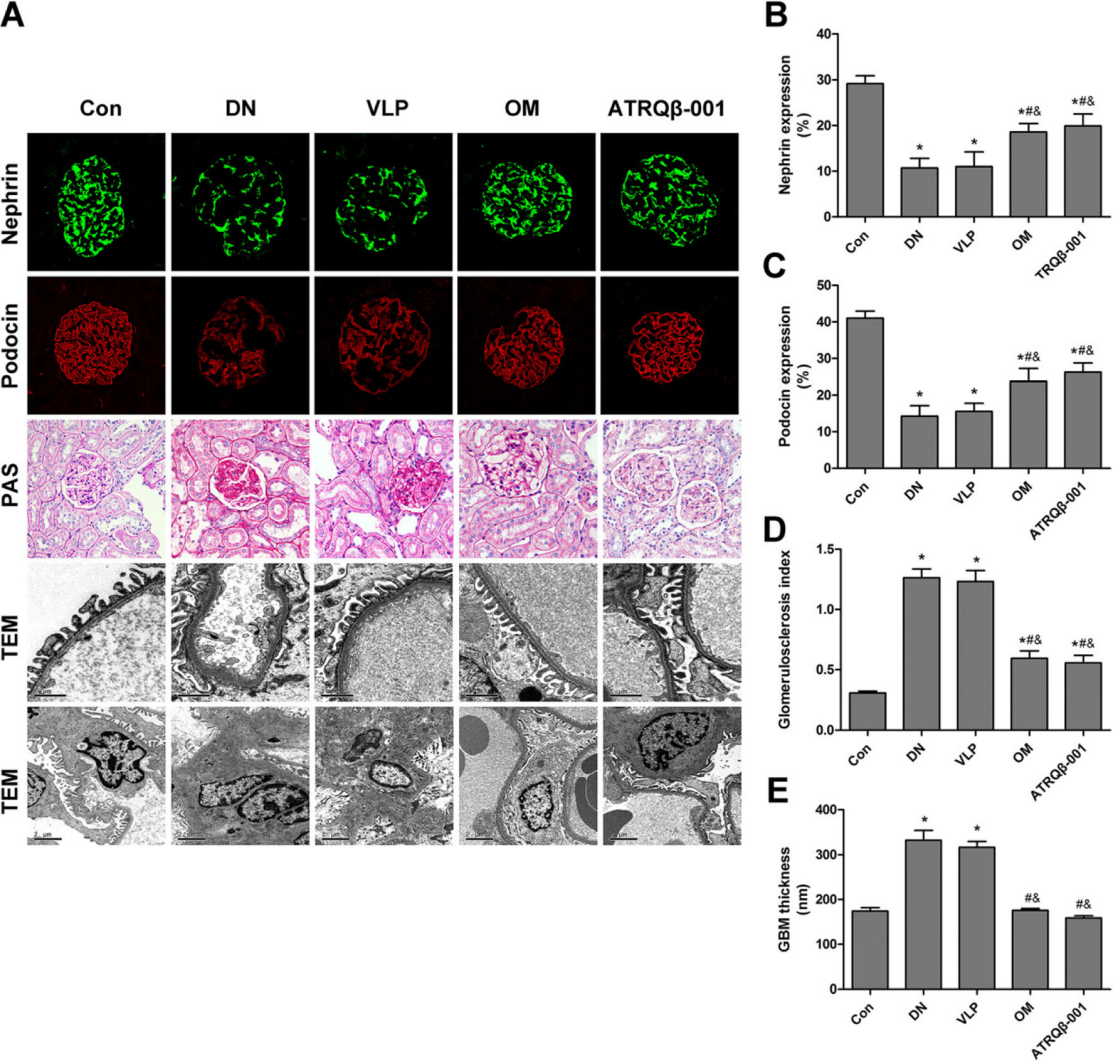
ATRQβ-001 vaccination prevented podocyte injury and loss as well as mesangial expansion. **a** Representative micrographs of nephrin and podocin expression, periodic acid-Schiff (PAS) staining (400×), and transmission electron microscopy (TEM). TEM showed the glomerular basement membrane and the mesangial area. **b** Quantitative analysis of nephrin expression. **c** Quantitative analysis of podocin expression. **d** Quantitative analysis of glomerular sclerosis index based on PAS staining. **e** Quantitative analysis of glomerular basement membrane thickness. *P < 0.05 vs. control group; #*P* < 0.05 vs. DN group; &*P* < 0.05 vs. VLP group

To confirm the effect of vaccination, we evaluated renal pathological changes. Podocytes form the filtration slit diaphragms that prevents the escape of plasma protein from the glomerular circulation. The disruption of podocytes contributes to proteinuria and further development of glomerular sclerosis [15]. Reduced nephrin and podocin immunostaining in glomeruli were showed in diabetic rats, and ATRQβ-001 vaccination prevented the reduction (Fig. 2).

In addition to podocyte injury, glomerular mesangial expansion is also a hallmark of DN. Extracellular matrix deposition was detected by periodic acid-Schiff staining, and glomerular sclerosis index (GSI) was calculated. Compared with DN and VLP groups, GSI was significantly decreased in ATRQβ-001-vaccinated group, similar with olmesartan-treated group, which was also confirmed by transmission electron microscopy (TEM) (Fig. 2).

### ATRQβ-001 vaccination ameliorated renal fibrosis and inflammation

**Fig. 3:**
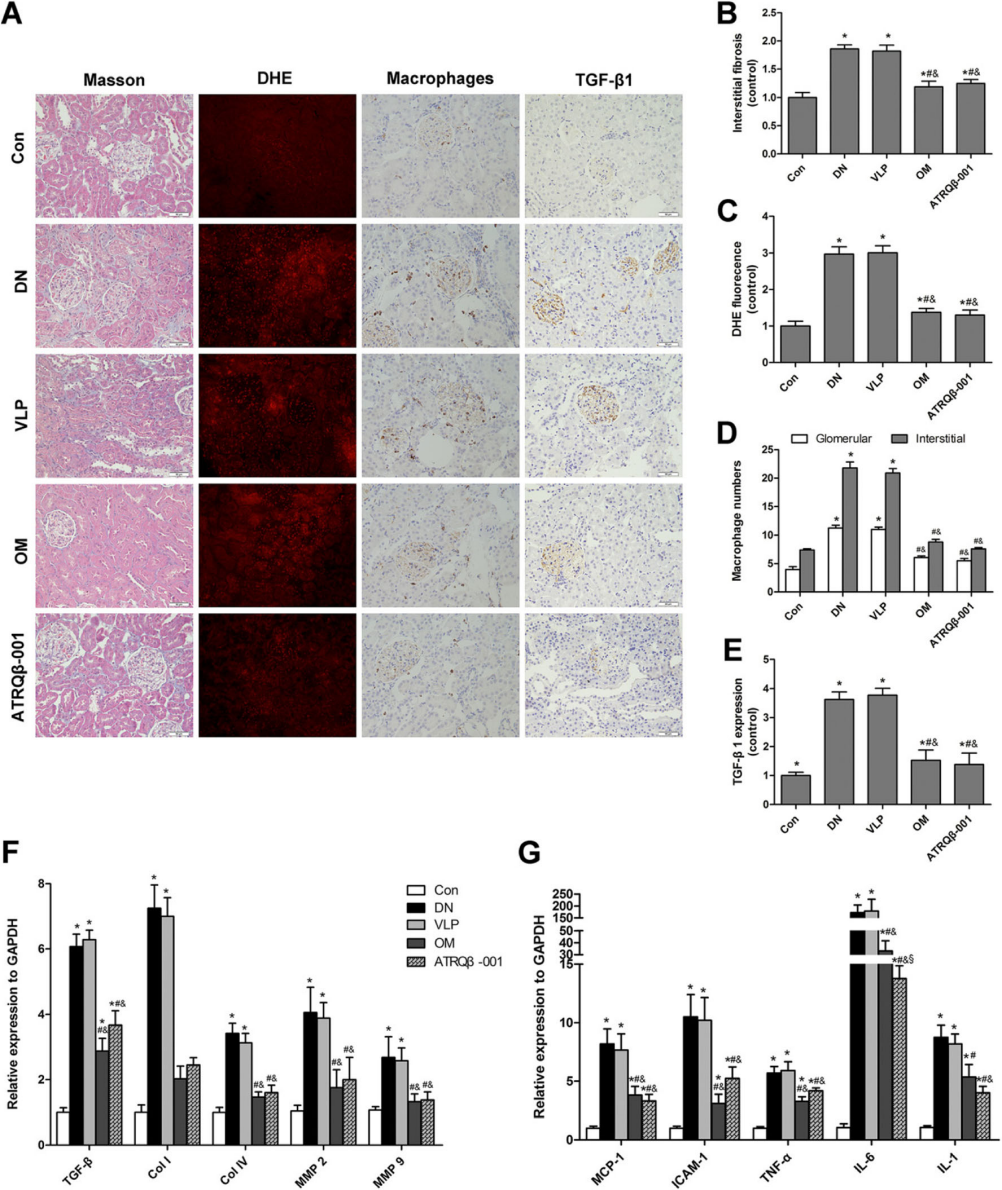
ATRQβ-001 vaccination ameliorated renal fibrosis and inflammation. **a** Representative staining of Masson’s trichrome, DHE (200×), macrophages, and TGF-β1 (400×). **b** Quantitative analysis of interstitial fibrosis. **c** Quantitative analysis of DHE fluorescence. **d** Quantitative analysis of glomerular and interstitial macrophage numbers. **e** Quantitative analysis of TGF-β1 expression. **f** The levels of profibrotic factor mRNA expression measured by quantitative real-time PCR (*n* = 10). **g** The levels of proinflammatory factor mRNA expression measured by quantitative real-time PCR (*n* = 10). *P < 0.05 vs. control group; #*P* < 0.05 vs. DN group; &*P* < 0.05 vs. VLP group

To further investigate the mechanisms, renal fibrosis and inflammation parameters were measured. The extent of interstitial expansion was quantified by Masson’s trichrome-stained sections. The interstitial expansive index was higher in DN and VLP groups, and ATRQβ-001 vaccination blocked the increase (Fig. 3). Glomerular and interstitial inflammation was quantified by the cell numbers of macrophages stained positively with CD68 antibody, and ATRQβ-001 vaccination successfully prevented the increased inflammation (Fig. 3). Besides, we detected transforming growth factor-β1 (TGF-β1) expression and reactive oxygen species, which was also decreased by ATRQβ-001 vaccine treatment. Further, the mRNA expressions of profibrotic and proinflammatory factors in kidney cortex were measured. As shown in Fig. 3f, g, the increased mRNA levels in these factors were significantly suppressed by ATRQβ-001 vaccination. There were no significant differences between ATRQβ-001- and olmesartan-treated groups.

### ATRQβ-001 vaccination suppressed renal Ang II-AT1R activation and abrogated the downregulation of ACE2-Ang (1–7) with no feedback of RAS

**Fig. 4:**
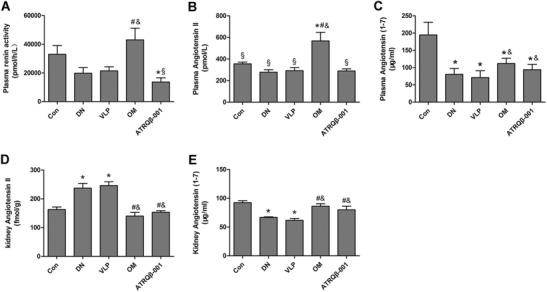
Circulating or local RAS were not activated in ATRQβ-001-vaccinated diabetic rats. **a** Plasma renin activity. **b** Plasma angiotensin II concentration. **c** Plasma angiotensin (1–7) concentration. **d** kidney angiotensin II concentration. **e** Kidney angiotensin (1–7) concentration. *N* = 10. *P < 0.05 vs. control group; #*P* < 0.05 vs. DN group; &*P* < 0.05 vs. VLP group; §*P* < 0.05 vs. OM group

To determine whether blockade of AT1R lead to feedback activation of circulating RAS, we detected the PRA and Ang II concentration. The PRA in ATRQβ-001 vaccinated group was 13 660 ± 2904 pmol/h/l, which had no significant difference with VLP group (21,450 ± 2851 pmol/h/l, *P* = 0.48). However, a distinct increase was observed in olmesartan group (43 040 ± 8 157 pmol/h/l, *P* = 0.005; Fig. 4a). Similarly, the plasma concentration of Ang II in olmesartan group was higher than VLP group (567.3 ± 80.7 vs. 291.8 ± 29.16 pmol/l, *P* = 0.02), whereas no significant difference was observed between VLP and vaccine groups (291.8 ± 29.16 vs. 288.7 ± 19.33, *P* = 0.53; Fig. 4b). To further examine the local RAS, kidney Ang II concentration was measured. The concentration of Ang II in kidneys was lower in olmsartan- and vaccine-treated groups compared to DN and VLP groups (Fig. 4d). To investigate whether the protection effect was a result of increase Ang (1–7) activation with simultaneous Ang II suppression, we subsequently measured plasma and kidney Ang (1–7). The decline of Ang (1–7) in diabetic rats was improved in ATRQβ-001 vaccine and olmesartan-treated groups (Fig. 4c, e).

**Fig. 5:**
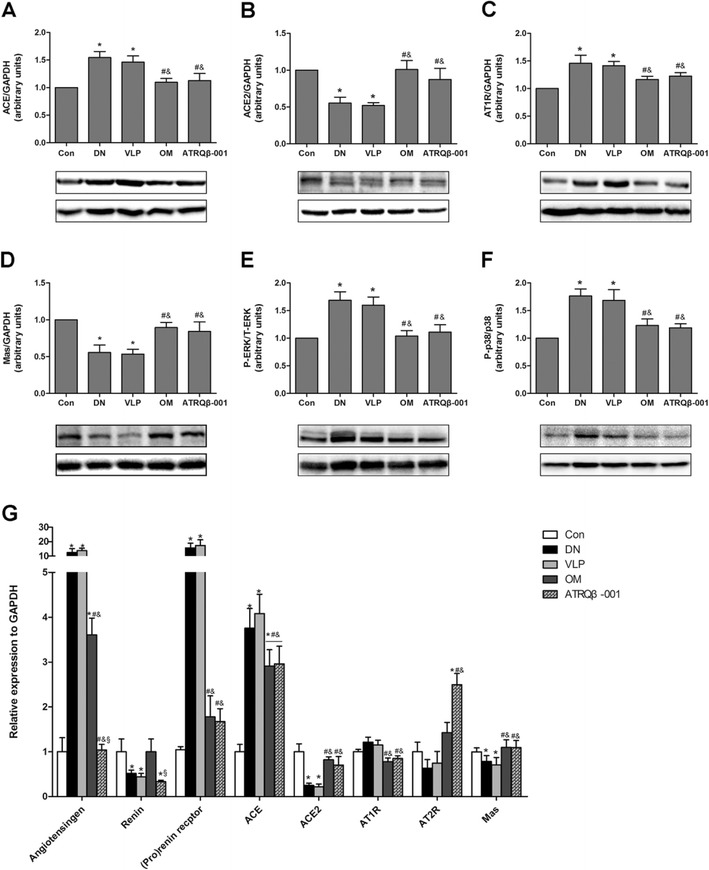
Effect of ATRQβ-001 vaccination on the RAS components, ERK1/2, and P38 MAPK in STZ-induced diabetic kidneys. Representative Western blots (*lower panel*) showing specific bands for **a** ACE, **b** ACE2, **c** AT1R, **d** Mas, **e** P-ERK1/2, and **f** P-p38 and quantitative analysis were presented on *upper panel* (*n* = 10). **g** The relative mRNA expression of angiotensingen, renin, (pro)renin receptor, ACE, ACE2, AT1R, AT2R, and Mas detected by quantitative real-time PCR (*n* = 10). *P < 0.05 vs. control group; #*P* < 0.05 vs. DN group; &*P* < 0.05 vs. VLP group

Activation of RAS in streptozotocin (STZ)-induced diabetic kidneys was further confirmed by Western blot and quantitative real-time PCR (Fig. 5). The mRNA expression of kidney renin in olmesartan group was obviously higher than VLP group (*P* < 0.001), while no difference was found between ATRQβ-001 vaccine and VLP groups (Fig. 5g). ACE and AT1R expressions were increased in diabetic rats, and both ATRQβ-001 vaccine and olmesartan groups attenuated the increasing expressions. ACE2-Ang (1–7)-mas axis counteract Ang II-mediated effects in various organs, including the kidneys, and we observed reduced expression of ACE2-Ang (1–7)-mas in the diabetic kidneys. ATRQβ-001 vaccine and olmesartan displayed upregulation of ACE2-Ang (1–7)-mas expression compared with VLP group. Further, we also detected the downstream signal transduction and the activation of ERK1/2, and p38 MAPK phosphorylation was inhibited by ATRQβ-001 vaccination, similar to olmesartan treatment (Fig. 5e–f).

### Anti-ATR-001 inhibited the TGF-β1/Smad3 signal pathway and expression of collagen IV and fibronectin in rat mesangial cells

**Fig. 6:**
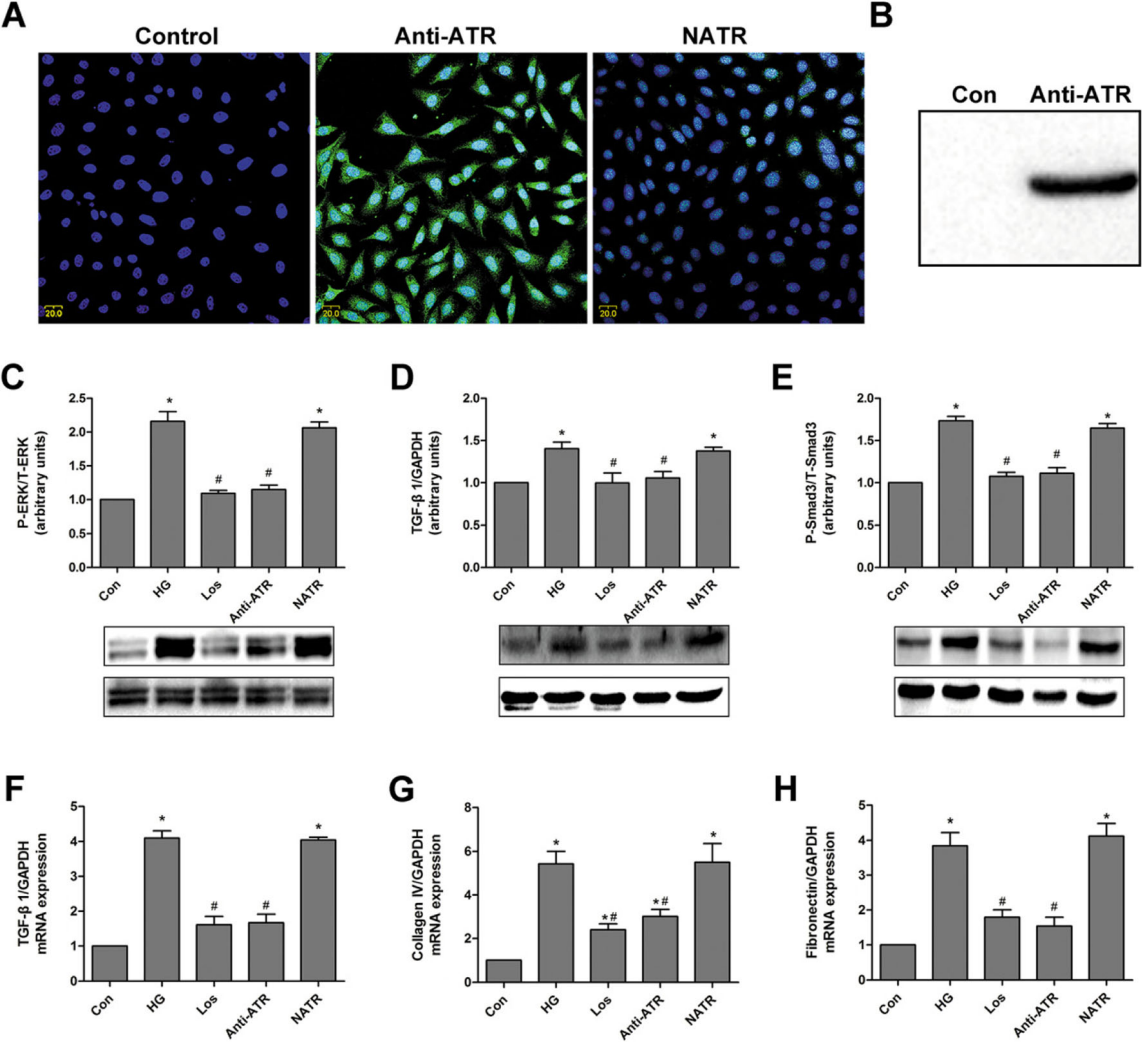
Anti-ATR-001 inhibited the TGF-β1/Smad3 signal pathway and expression of collagen IV and fibronectin in rat mesangial cells. As shown on **a** and **b**, anti-ATR-001 specifically bound to AT1R in rat mesangial cells (RMCs). Representative Western blots (*lower panel*) showing specific bands for **c** P-ERK1/2, **d** TGF-β1, **e** Smad3, and quantitative analysis were presented on *upper panel*. The relative expression of TGF-β1 and collagen I and IV mRNA were shown on **f**, **g**, and **h**. *Con control*, HG high glucose (25 mmol/l), *Los* losartan treatment, *Anti-ATR* anti-ATR-001 treatment, *NATR* anti-NATR-001 treatment (*n* = 5). *P < 0.05 vs. con; #*P* < 0.05 vs. HG

To further explore the mechanisms, cell experiment was undertaken by anti-ATR-001 stimulation. Glomerular mesangial cells are believed to be responsible for overproduction of ECM proteins in various pathologic conditions, and TGF-β1 has been proposed to play an important role. The TGF-β1/Smad3 signal pathway regulates the hypertrophic and prosclerotic changes in diabetic renal injury [16]. Anti-ATR-001 and losartan treatment significantly decreased the expression of TGF-β1 and the phosphorylation levels of Smad3 in RMCs stimulated by high glucose. Additionally, we examined the expression of collagen IV and fibronectin, which showed similar change as TGF-β1 (Fig. 6).

### No imumune-mediated injury was observed in vaccinated animals

**Fig. 7:**
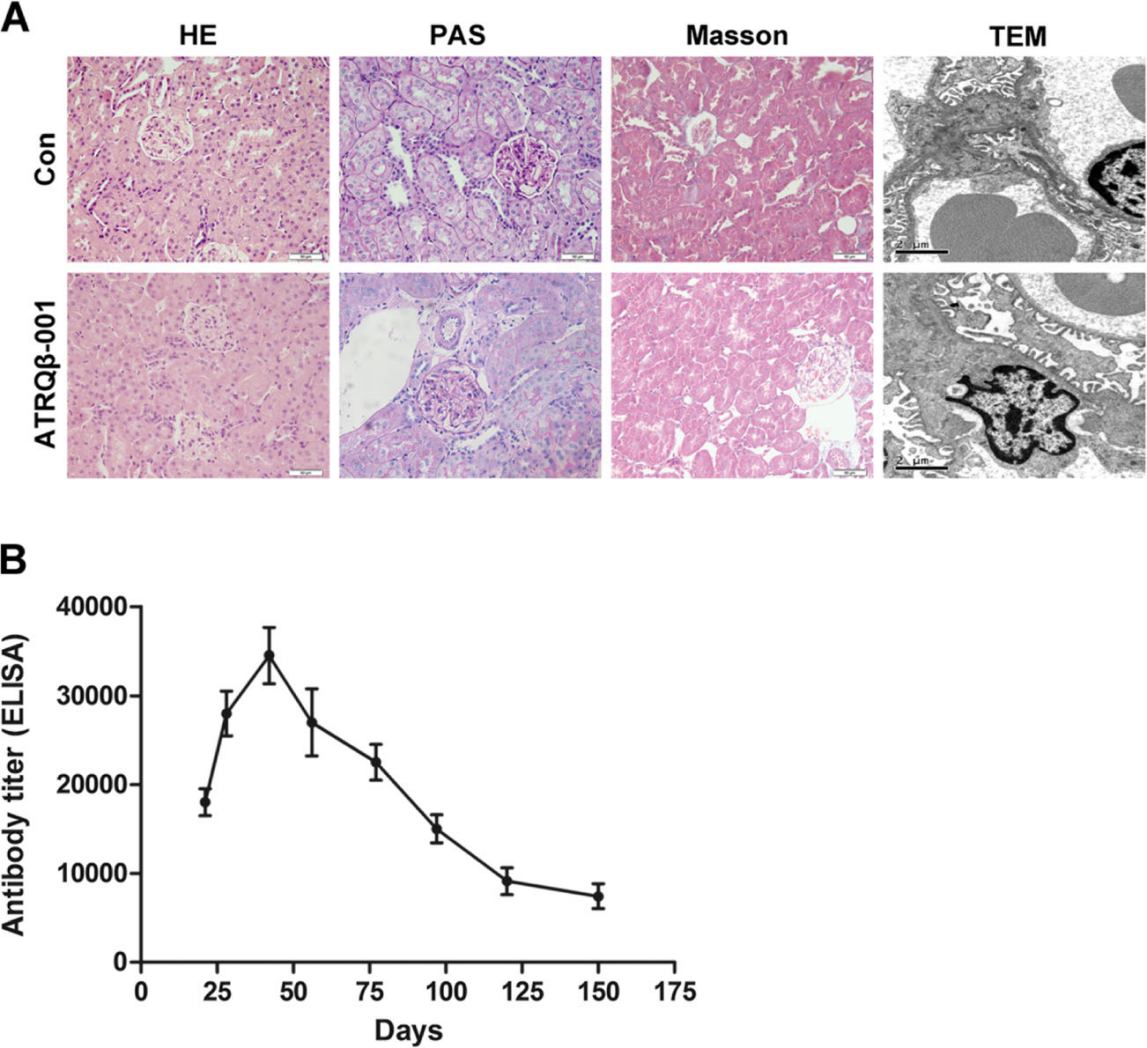
No imumune-mediated injury was observed in vaccinated animals. **a** Representative staining of hematoxylin-eosin (H&E), Masson’s trichrome, and periodic acid-Schiff (PAS) and transmission electron microscopy (TEM). **b** Normal SD rats were immunized on days 0 and 14, and the ATR-001-specific antibody titers were screened on days 21, 28, 42, 56, 77, 97, 120, and 150

For safety consideration, normal SD rats were immunized with the ATRQβ-001 vaccine, and the histological changes of kidney were observed by light microscopy and TEM. No evidence of skin damages at the site of subcutaneous injection was noted in vaccine-treated animals. Compared with control group, no obvious cell proliferation and pathological changes in the mesangial area were shown in vaccine group. Also, TEM demonstrated that no immune complexes were observed in the basement membrane, and the structure of the glomerulus was intact (Fig. 7).

## Discussion

In this study, we demonstrated for the first time that in a rat model of DN, a vaccine targeting AT1R named ATRQβ-001 attenuated the progression of the disease, as exhibited by reduction of biochemical parameters of renal dysfunction and amelioration of renal pathological changes.

The rat DN model was induced by an intraperitoneal injection of STZ that has been widely used to create diabetes models in rodents with pathological features similar to human diabetic nephropathy [14]. The nephropathy subcommittee of the Animal Models of Diabetic Complications Consortium (AMDCC) has published the following validation criteria for rodent models of DN based on the clinical and pathological features of human DN: (1) >50 % decrease in renal function, (2) >10-fold increase in albuminuria, and (3) pathological features including advanced mesangial matrix expansion (±nodules), thickening of the glomerular basement membrane, arteriolar hyalinosis, and tubulointerstitial fibrosis [17]. An ideal model of DN would display all of these criteria; however, no current model entirely satisfies them. That is the limitation in animal experiments. As the rodent models share many similarities with human disease, we cannot ignore the potential therapeutic value of the vaccine in the treatment of human diseases. Our results showed that rats presented severe hyperglycemia and renal dysfunction manifested as increased plasma creatinine level, BUN, 24 h urinary volume, and protein excretion, indicating that a DN animal model was successfully established. The ratio of kidney weight/body weight in diabetic groups was significantly increased compared with that in control group, substantial to the presence of renal hypertrophy in diabetic rats. During the course of the study, systolic blood pressure was elevated in diabetic rats, notwithstanding the limitations of tail-cuff sphygmomanometry, ATRQβ-001 vaccination lowered blood pressure (BP) smoothly, and olmesartan decreased to a greater extend. It is worth noting that our animal experiments showed many times that the antihypertensive effect of ATRQβ-001 vaccine was dependent on the baseline of the BP level and exhibited no decreasing effect in normotensive rats. Oppositely, ARBs decreased BP irrespective of the basic level and had an increase risk of hypotension. Therefore, ATRQβ-001 vaccine may be more practical in diseases where the BP was not very high, such as DN.

Glomerular mesangial matrix expansion, renal fibrosis, and inflammation are typical pathological features of DN. ATRQβ-001 vaccination ameliorated these morphologic changes of renal injury as well as decreased expression of profibrotic and proinflammatory factors. To further confirm the effects, RMCs were used to investigate the mechanism in vitro. The anti-ATR-001 antibody treatment effectively inhibited high glucose-induced extracellular signal-regulated kinase phosphorylation and TGF-β1/Smad3 signal pathway. These results strengthened the renoprotection of ATRQβ-001 vaccination in addition to the antihypertensive effect. The inhibition of oxidative stress and macrophage infiltration as well as proinflammatory factor expression may contribute to the amelioration of renal pathological changes.

Furthermore, compared with the obvious RAS feedback of ARBs, circulating or local RAS were not elevated in diabetes animals immunized with ATRQβ-001 vaccine. Nevertheless, kidney Ang II concentration and AT1R expression were decreased in both groups. The recent development of ACE2-Ang (1–7)-mas concept has provided new insights into the effects of Ang II in animal experimental models. This novel concept states that RAS has two axes: the ACE-Ang II-AT1R axis and ACE2-Ang (1–7)-mas axis. The former axis induces vasoconstriction, proliferation, and proinflammatory functions through Ang II, and the latter axis counteract the effects of the former axis through the major heptapeptide effector Ang (1–7) [18, 19]. ACE2 cleaves Ang II to produce abundant levels of Ang (1–7) in the proximal tubule, which produces vasodilation and antiproliferative, natriretic, and diuretic effects [20]. Despite its significant role, the exact role of ACE2 and Ang (1–7) in kidney disease is not clearly understood. It has been reported that inhibition of ACE2 function accelerates diabetic injury and human recombinant ACE2 reduces the progression [21, 22]. Moreover, Ang (1–7) attenuates the progression of diabetic nephropathy in animal models [23, 24]. Our study showed a downregulation of ACE2-Ang (1–7)-mas axis in diabetic rats, and the beneficial effects of ATRQβ-001 vaccine and olmesartan treatment may partly due to the upregulation of this axis. Although the exact mechanism of how these two axes counteract with each other remains unknown, we can conclude that the vaccine regulates the two RAS axes, not only inhibiting AT1R overactivation.

Safety considerations are paramount when developing any vaccine but are particularly important when targeting a condition for which many safe and effective alternative therapies are available. Four key factors define the safety of therapeutic vaccine: (1) the targeted molecule, (2) the reversible of antibody response, (3) antibody-dependent cell-mediated cytotoxicity (ADCC) and complement-mediated cytotoxicity, and (4) activation of T cells against self-molecules [25]. Combined with our previous studies, vaccination-targeted AT1R showed no immune-mediated injuries [11, 12] and was also confirmed in this study. The antibody response was reversible as the possibility of halting antibody production. For ADCC and complement-mediated cytotoxicity, there are still several controversies in terms of its onset and regulation. Nevertheless, the potential effects caused by the vaccine needed further investigation. As the target peptide of the vaccine was only eight amino acids in length, then it was smaller than the minimal size of a T cell epitope and therefore should not be able to induce a T cell response [26]. Several studies had estimated that vaccines comprising self-molecules coupled to VLPs present optical candidates capable of achieving efficacious antibody levels while fulfilling the necessary safety criteria [27, 28]. Examples like the Ang II vaccine CYT-006-AngQb, which showed effectively reduced BP without any uncontrolled immune stimulation in both animals and patients [29, 30]. Similar to the composition, the kidney damage caused by immune complexes was not detected and no visible pathological changes were observed by light microscopy and TEM in ATRQβ-001-vaccinated animals. From the results above, the ATRQβ-001 vaccine was found to be basically safe, although further assessments are needed to confirm this conclusion.

Compared with chemical drugs, the vaccine therapy has potentially superior advantages. The half-life of the anti-ATR-001 antibody was 14.4 days [11], longer than any other chemical drugs presently used. No obvious feedback activation of circulating or local RAS was observed. Additionally, long-term treatment of chronic diseases is costly, tedious, and at the population level rather unsuccessful. Monoclonal antibodies specific for host proteins have proven to be highly effective, and the movement from the passive administration of monoclonal antibodies to active vaccination against self-molecular could provide affordable medicines and broader patient acceptance and compliance. The question today in many patient populations is not whether to block the RAS but rather how best to inhibit its activity. From a theoretical perspective, vaccination has much to recommend it as a strategy. ATRQβ-001 vaccine modulates two major axes of RAS and ameliorates STZ-induced diabetic injury with no obvious immune-mediated damages, providing a promising therapeutic method to treat diabetic nephropathy.

## Electronic supplementary material

Below is the link to the electronic supplementary material.

ESM 1 (DOC 79 kb)
