# Supplementary material for: Vaccination against type 1 angiotensin receptor prevents streptozotocin-induced diabetic nephropathy
Source: J Mol Med (Berl). 2015 Sep 26;94(2):207–18. doi: 10.1007/s00109-015-1343-6 (PMC4762923; doi:10.1007/s00109-015-1343-6)
Supplement: Supplementary file 1 — (DOC 79 kb) [file 109_2015_1343_MOESM1_ESM.doc]

**ELECTRONIC SUPPLEMENTARY MATERIALS:**

**Vaccination against type 1 angiotensin receptor prevents**

**streptozotocin-induced diabetic nephropathy**

Dan Ding, Yimei Du, Zhihua Qiu et al.

*J Mol Med 2015*

**__________________**

**Materials and Methods**

**Peptide synthesis**

The ATR-001 peptide, with the sequence C-A-F-H-Y-E-S-Q corresponding to an epitope of the ECL2 of human AT1R, was synthesized by GL Ltd. (Shanghai, China). The purity was above 98% and was determined using high-performance liquid chromatography (HPLC) and mass spectrometry (MS).

**Vaccine preparation**

The Qβ VLP prokaryotic expression plasmid was constructed, expressed in BL21 *Escherichia coli* and purified by acidification, sedimentation of saturation ammonium sulfate, hydrophobic interaction chromatography (GE Healthcare), and gel filtration chromatography (GE Healthcare). The expressed VLP was identified using sodium dodecylsulfate-polyacrylamide gel electrophoresis (SDS-PAGE) and transmission electron microscope (TEM). The ATR-001 peptide was covalently conjugated to VLPs using the Sulfo-SMCC cross-linker (Pierce) to produce the ATRQβ-001 vaccine. The vaccine concentration was determined using Bradford protein assay kit (Pierce).

**Animals**

Male Sprague Dawley rats weighing 200-250g were purchased from the experimental animal research center (Hubei province, China). All animals were kept in the pathogen-free room in the experimental animal center (Tongji Medical College of Huazhong University of Science and Technology, Wuhan, China), and all experiments were carried out in accordance with guidelines for the Care and Use of Laboratory Animals (Science and Technology Department of Hubei Province, China, 2005). Experimental diabetes was induced by intraperitoneal injection of the β-cell toxin streptozocin (60 mg/kg) dissolved in fresh sodium citrate buffer (pH 4.5) following an overnight fast. Animals with plasma glucose concentrations in excess of 16.7 mmol/l, 1 week postinduction of diabetes, were included in the study. Sham-injected control animals (sodium citrate buffer, pH 4.5) were followed concurrently. Diabetes rats were then randomized into four groups(n=15), receiving one of the following treatments: (1) DN group: equal volume saline injection subcutaneously (s.c); (2) OM group: the angiotensin Ⅱ type 1 receptor blocker, olmesartan, 5mg/kg/d via oral gavage; (3) ATRQβ-001group: the ATRQβ-001 vaccine, which was immunized s.c 400ug on days 0, 14 and 21, formulated in aluminum hydroxide gel; (4) VLP group: 400ug Qβ VLP injected as group 3. Each week, rats were weighed and their blood glucose levels were measured. ATR-001-specific antibody titers were detected in every two weeks. Every four weeks, systolic blood pressure (SBP) was determined in preheated conscious rats via tail-cuff plethysmography using a non-invasive blood pressure controller and Powerlab system.

**Plasma renin activity (PRA), Ang Ⅱ and Ang (1-7) concentration measurement**

The rats were decapitated between 9 am and 12 am. Blood samples were collected and divided into two parts: one was mixed with the enzyme inhibitor mixture (1 ml blood in 50 μl inhibitor mixture including 20 μl 0.3 mol/L EDTA, 10 μl 0.32 mol/L dimercaprol and 20 μl 0.34 mol/L 8-OH-quinoline sulphate) for PRA and Ang Ⅱ concentration measurement by radioimmunoassay (RIA) according to the assay kit instruction (NIBT, Beijing); the other was used for biochemical experiments. For tissue Ang Ⅱ and Ang (1-7) measurement, immediately after harvesting and weighing, the kidney cortex were immersed in ice-cold methanol, minced, and homogenized with tissue homogenizers. The homogenates were centrifuged (4000rpm, 4 ℃, 15min) and the supernatants were dried overnight in a vacuum centrifuge. The dried residue was reconstituted in 1 ml radio-immunoassay (RIA) buffer and then was subjected to HPLC to separate Ang Ⅱ from other substances. The tissue Ang Ⅱ concentration was detected according to the assay kit instruction (NIBT, Beijing) and Ang (1-7) was measured by HPLC.

**Biochemical measurement**

The plasma samples were used for the measurement of glucose, lipids level, creatinine (Cr), blood urea nitrogen (BUN). Urine samples were collected using metabolic cages and the supernatant was used for examination of the 24-hour urinary protein.

**Histopathology**

Kidneys were quickly removed and weighed. The renal mass index was calculated as the ratio of the weight of both kidneys to body weight. The right kidneys were stored in a -80 ℃ freezer for biochemistry. Parts of fresh left renal cortex were immediately fixed in 0.25% glutaraldehyde for transmission electron microscopy (TEM). The other parts were ﬁxed with 4% paraformaldehyde overnight, embedded in paraffin, and cut into 4-μm sections for histopathology. Sections were stained with hematoxylin-eosin (H&E), Masson’s trichrome and periodic acid-Schiff (PAS). Frozen sections were stained with DHE.

**Immunohistochemistry**

Immumohistochemical staining was performed to detect the expression of TGF-β1 (1:100, Everest Biotech) and macrophages (CD68, 1:100, AbD Serotec) in glomeruli.

**Immunofluorescence**

Immunofluorescence staining of nephrin and podocin expression were performed on the paraffin sections of kidneys using monoclonal anti-nephrin (1:200, Abcam) and anti-podocin antibodies (1:200, Abcam).

**Quantitative real-time PCR (qRT-PCR)**

Total RNA was extracted from renal cortex by Trizol reagent (Invitrogen, California, USA). Single-stranded cDNA was produced by reverse transcription with the Reverse Transcriptase kit (Takara, Japan). SYBR Premix Ex Taq^TM^ Ⅱ (Takara, Japan) and the Applied 7000 (Applied Biosystem) were used for quantitative real-time PCR. The primers were shown in Table S1. Samples were amplified in duplicate for 40 cycles and target gene expression was normalized to the expression of GAPDH.

**Western blot**

The total proteins of the heart tissues were extracted with the Total Protein Extract ion Kit (Pierce/Thermo Scientific, Rockford, IL). Protein concent- ration was determined by the BCA Protein Assay Kit (Pierce). Samples were separated on a 10% SDS-PAGE and electrotransferred onto nitrocellulose membranes. Membranes were sequentially blocked in TBST containing 5% skim milk and then incubated with primary Abs against ACE (1:1000, Abcam), ACE2 (1:200, Abcam), AT1R (1:500, Abcam), masR (1:1000, Novus Biologicals), P-ERK1/2 (1:1000, Cell Signaling technology), T-ERK1/2 (1:1000, Cell Signaling Technology), P-p38 MAPK (1:1000, Cell Signaling Technology), p38 MAPK (1:1000, Cell Signaling Technology) and GAPDH (1:1000, Cell Signaling Technology) at 4°C overnight. After washing, the membranes were then further incubated with HRP-conjugated secondary Ab (1:3000, 37°C, 2 h). The target bands were finally washed and developed with super ECL reagent (Thermo Scientific) and semiquantitatively analyzed using densitometric methods.

**Antibody preparation**

New Zealand white rabbits were immunized with ATR-001 peptide that was conjugated to keyhole limpet hemocyanin (KLH). After three subcutaneous injections, the rabbits were placed on a controlled heating pad and injected with buprenorphine hydrochloride (0.2mg/kg) subcutaneously for analgesia. The rabbits were anesthetized with an intravenous injection of pentobarbital sodium (50 mg/kg). Heparin (2000 IU/kg) was used as an anticoagulant. The heart rates, breathing rates, color of the mucosa and autonomic reflexes were monitored. Blood was collected by cardiac puncture when the palpebral reflex was lost, and the method of euthanasia was exsanguination of the anesthetized rabbits. The antibody titers were determined using an enzyme-linked immunosorbent assay (ELISA). The serum was purified using protein A affinity chromatography (Bio-Rad). The antibodies against ATR-001 were termed Anti-ATR-001, respectively. Both antibodies were additionally purified by epitope-linked gel affinity chromatography (GE Healthcare), and the concentrations were confirmed by BCA kit (Pierce). The ‘Control Antibody’ was purified from rabbits that were immunized with KLH only. In all of the experiments, the final working concentration of the antibody was 1.0 μg/ml. The peptide neutralization antibodies were produced by the co-incubation of 1 mg of each peptide and 1 mg of the respective antibody in phosphate-buffered solution (PBS; 137 mmol/L NaCl, 2.7 mmol/L KCl, 4.3 mmol/L Na_2_HPO_4_, 1.4 mmol/L KH_2_PO_4_, PH 7.4) for 8 h at 4 °C. The peptide neutralization antibodies were termed the NATR-001 antibody. The final working concentrations of the NATR-001 antibodies were 1.0 μg/ml.

**Cell culture and treatment**

Rat mesangial cells (RMCs) were purchased from the American Type Culture Collection and cultured in Dulbecco’s modified Eagle’s medium supplemented with 10% fetal bovine serum, 100 U/ml penicillin, and 100mg/ml streptomycin at 37 ℃ in 95% air and 5% CO_2_. Cells plated on 60-mm dishes were cultured to 80% confluence and divided into five groups: Control group, in which cells were incubated in 5 mmol/l D-glucose DMEM, and 20 mmol/l D-mannitol was added to the medium in order to take into the account of the effect of high osmolarity in other cell groups; HG group, in which cells were stimulated with a high concentration of glucose (25 mmol/l) only for 24 h; Los group, in which cells were pretreated with 10^-6^ mol/l losartan for 1 h; Anti-ATR-001 group, in which cells were pretreated with anti-ATR-001 for 1 hour; Anti-NATR-001 group, in which cells were pretreated with anti-NATR-001 for 1 hour. All cells except control group received stimulation with a high concentration of glucose (25 mmol/l) for 24 h after treatment. Finally, cell protein and mRNA were extracted for western blot and qRT-PCR analysis,

**Statistical analysis**

Data were shown as the mean + SEM. Statistical analyses of the data were performed with one-way ANOVA using SPSS18.0. P<0.05 was considered statistically significant.

**Supplement Table**

**Table S1. Sequences of primers for quantitative real-time PCR**

| **Molecules** | | **Sequence(5'-3')** |
| --- | --- | --- |
| Renin | sense | GGGTGCTAAAGGAGGAAGTGTT |
|  | anti-sense | GTGAAAGTTGCCCTGGTAATGT |
| Angiotensinogen | sense | CCCGTGGATGAGAAGACCC |
|  | anti-sense | GATGCGGAAACCCATGAAG |
| ACE | sense | CCGTGTTGCCAATGACATAGAA |
|  | anti-sense | GCACCAGTCGTAGTTGTAGCG |
| ACE2 | sense | CGTGTTGCCAATGACATAGAA |
|  | anti-sense | GCACCAGTCGTAGTTGTAGCG |
| AT1R | sense | CATCGTCCACCCAATGAAGTC |
|  | anti-sense | AGGGAACAAGAAGCCCAGAAT |
| AT2R | sense | AATCTGGCTGTGGCTGACTT |
|  | anti-sense | GCTTCTGACATCTCGGAAATAA |
| PRR | sense | GAGGCAGTGACCCTCAACAT |
|  | anti-sense | CCCTCCTCACACAACAAGGT |
| Mas | sense | GACCAGCCCACAGTTACCAGTT |
|  | anti-sense | TTCCGGCTTTCTGGATTCTCA |
| Col Ⅰ | sense | TCCGAGCCAGTCCGTTTAT |
|  | anti-sense | ACACCTGACAGCGGCTTATG |
| Col Ⅳ | sense | ATTCCTTTGTGATGCACACCAG |
|  | anti-sense | AAGCTGTAAGCATTCGCGTAGTA |
| MMP 2 | sense | TTGGAAGCATCAAATCGGACTG |
|  | anti-sense | CCACCCTCTTAAATCTGAAATCACC |
| MMP 9 | sense | GCAAACCCTGCGTATTTCCATT |
|  | anti-sense | AGCCATACAGCTTATCCTGGTCAT |
| Fibronectin | sense | GCCTGGTACAGAATATGTAGTG |
|  | anti-sense | ATCCCAGCTGATCAGTAGGCTGGTG |
| TGF-β1 | sense | CCAAGGAGACGGAATACAGGG |
|  | anti-sense | GCTATGAGGAGCAGGAAGGGT |
| ICAM-1 | sense | GCGTGAACTTGACCCATAAATC |
|  | anti-sense | GGCTTGGAAGGGAATAGTGTAAT |
| IL-1 | sense | GCTTCAAATCTCACAGCAGCATCT |
|  | anti-sense | CTCCACGGGCAAGACATAGGTAG |
| IL-6 | sense | GACAGCCACTGCCTTCCCTACT |
|  | anti-sense | GCATTGCCATTGCACAACTCTT |
| MCP-1 | sense | CTCTCTTCCTCCACCACTATGC |
|  | anti-sense | ACTACAGCTTCTTTGGGACACC |
| GAPDH | sense | CTCCCTCAAGATTGTCAGCA |
|  | anti-sense | GTTCAGCTCTGGGATGACCT |
